# Benchmarking retrieval augmented generation LLMs for Arabic noise robustness

**DOI:** 10.3389/fdata.2026.1884673

**Published:** 2026-08-17

**Authors:** Saleh Almohaimeed, Abdulrahman Alabduljabbar, Mousa Jari, Mohammed Alkhowaiter, Saad Almohaimeed, Mohamad Mahmoud Al Rahhal

**Affiliations:** 1 Department of Applied Computer Science, College of Applied Computer Science, King Saud University, Riyadh, Saudi Arabia; 2 Department of Computer Engineering, College of Computer Engineering and Sciences, Prince Sattam Bin Abdulaziz University, Al-Kharj, Saudi Arabia; 3 Department of Digital Transformation, Institute of Public Administration, Riyadh, Saudi Arabia

**Keywords:** Arabic natural language processing, large language models, LLM noise robustness, question answering systems, retrieval-augmented generation

## Abstract

Hallucination has become a serious concern in large language models (LLMs), as these models can generate useful yet incorrect or misleading information, which has led to growing research interest in retrieval-augmented generation (RAG) as a mitigation approach. RAG provides LLMs with access to external information, such as databases or documents, which can help them to answer users' questions. Currently, several benchmarks released measure RAG performance on various LLMs; however, evaluations of the noise robustness ability in Arabic are absent. In this paper, we systematically investigated the capabilities of state-of-the-art multilingual LLMs with regard to two essential RAG abilities, noise robustness and negative rejection. To accomplish this, we generated an Arabic benchmark consisting of 300 questions along with 6,196 documents. Then, we assessed the performance of six LLMs in relation to the two aforementioned RAG abilities. The results reveal that all six LLMs were negatively affected when the noise ratio in the external documents was increased. Under the highest noise settings at 80%, the best LLM performance was for Claude-4 sonnet, in which their performance decreased by only 4.67 percentage points. Furthermore, when it comes to the negative rejection task, there has been a significant impact on all six models. The best two models, Claude-4 sonnet and Llama-4, scored 90.67% and 85.67%, respectively, while smaller models, like GPT-3.5, scored 69.33%. Furthermore, our manual analysis reveals that many errors made by LLMs are attributed to over-caution behavior. LLMs often decline to respond probably due to training mechanisms designed to reduce hallucinations. Additionally, other errors occur when there is a high lexical similarity between the question and the words of noisy documents, which causes the model to rely on irrelevant content instead of the correct information.

## 1 Introduction

Artificial intelligence (AI) has made significant progress over the past few years. Regular users have been using popular LLMs most often, and some of them rely on LLMs output as facts. However, when it comes to generating content, these LLMs experience a great deal of hallucination (Shuster et al., 2021). This is due to the fact that some input requires domain knowledge, comprehension of the prompt, and up-to-date information. In addition to the content generation hallucinations, LLMs also suffer from multilingual limitations (Romanou and Foroutan, 2024; Chua et al., 2024), and design constraints (Shen and Tan, 2024) that prevent them from producing the correct content.

**Figure 1:**
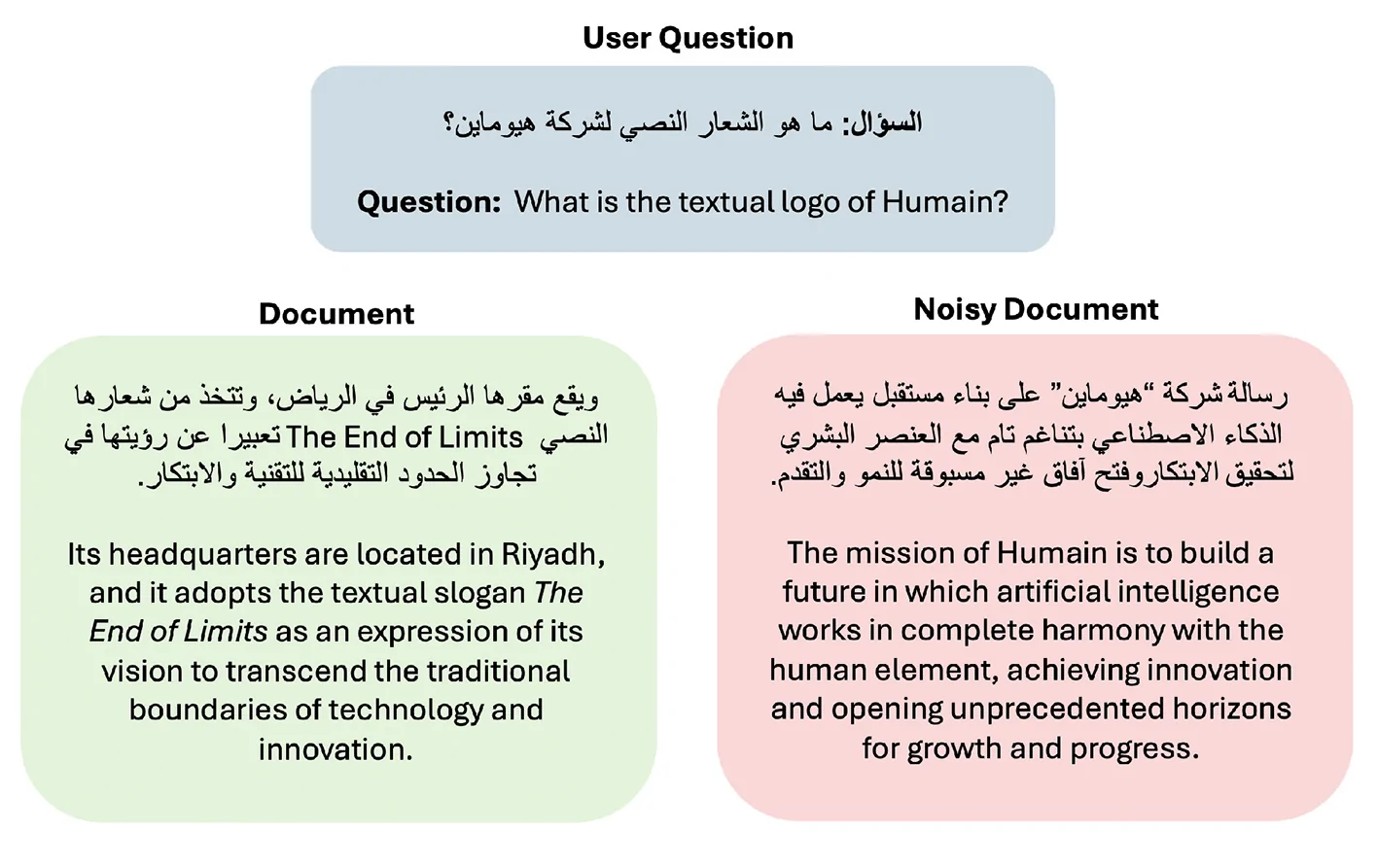
An example of a user's question with two documents. The left document contains the correct answer, while the right document discusses the company mentioned in the question without providing an answer. The second document is considered as a noisy document.

For these reasons, a new field in AI has emerged named Retrieval-Augmented Generation (RAG) (Lewis et al., 2020a; Borgeaud et al., 2022; Guu et al., 2020), which uses external documents along with LLM knowledge to answer the user's question. In such a way, it brings in-domain and up-to-date information, which mitigates the effect of hallucinations on LLMs. To do that, most LLMs utilize a search engine API to search the internet and retrieve relevant documents, which are then incorporated into the user prompt. However, some of these documents could be irrelevant or contain noise information, as illustrated in Figure 1. An evaluation for such a task is needed and understanding the capabilities of LLMs to distinguish between relevant and irrelevant documents is essential.

Some efforts in English and Chinese have been made in Chen et al. (2024), where they have demonstrated that a model like ChatGPT (OpenAI, 2022) in answering English questions without noise documents got a percentage of 96.33%. However, when many noisy documents were included, the performance decreased significantly to 76.00%. Similarly, in answering Chinese questions, they got 95.67% without noise documents, and performance declined to 70.67% with many noise documents. There are not many efforts made in other languages like Arabic, where they study the effect of noise documents on Arabic RAG systems.

In this paper, we have performed a comprehensive evaluation of six popular LLMs, including GPT-4o (Hurst et al., 2024), GPT-3.5 (OpenAI, 2024), Claude-4 sonnet (Anthropic, 2025b), Llama-4 (Meta AI, 2025), Llama-3 (AI, 2024), and DeepSeek-V3 (DeepSeek-AI et al., 2024) on two tasks. The first task is noise robustness (Amiraz et al., 2025; Wang et al., 2025; Abdaljalil et al., 2025b), which studies the effect of noise documents on the response of LLMs. The second task is negative rejection (Chen et al., 2024; Abdaljalil et al., 2025c), which examines whether LLMs can accurately indicate the absence of an answer in scenarios where external documents are entirely noisy and do not contain the correct answer. To do both tasks, we have generated an up-to-date dataset with questions and answers related to an event that happened after the release date of the six experimented LLMs, this ensures that none of the LLMs could have encountered our questions during their training phase. Then we have tested the LLMs for noise robustness under six different settings (0%, 20%, 40%, 60%, 80%, 100%). On this basis, we can understand how these LLMs will stand against different levels of noisy documents. For all settings except the last one 100%, we are looking that the model get the right answer. However, under 100% setting of noise documents, this is related to the second task, which is negative rejection.

The results reveal that for noise robustness, the best model is the Claude-4 sonnet (Anthropic, 2025b), which achieved 91.67% under 0% noise documents and decreased to 87% under 80% noise documents, indicating robustness performance that is not adversely affected by additional unrelated information. In contrast, one of the worst models was GPT-4o (Hurst et al., 2024), despite showing remarkable results in different Arabic tasks (Alsulami and Almansour, 2025; Almohaimeed et al., 2025a; Al Wazrah et al., 2025), in regard to just answering the question without even noise documents, it achieved 74.33%. In many cases, the responses were “The documents do not contain the necessary information.” Even when the answer was presented in the given documents, GPT-4o sometimes responded with such a statement. There is a possibility that the model adopted a more cautious response strategy when faced with ambiguity. However, our experiments do not directly examine the internal mechanisms responsible for these responses.

Moreover, as indicated in the prompt instructions, if the model fails to locate the answer, it must indicate that the documents do not contain the necessary information. Thus, whenever GPT-4o encounters any ambiguity, it reports this uncertainty and behaves with caution. On the other hand, when it comes to negative rejection, the best models are Claude-4 sonnet, Llama-4 and GPT-4o, which achieve 90.67 %, 85.67 %, and 80.67 %, respectively. In contrast, the GPT-3.5 showed very poor performance achieving only 69.33 %. Possibly this is one of the reasons why GPT-4o performs more cautiously on the noise robustness task, as the previous OpenAI (OpenAI, 2025) model, GPT-3.5, suffered from severe factual hallucinations.

To the best of our knowledge, we are the first to study the effects of noisy documents on LLMs when answering Arabic questions. The contributions of our work are:

- Construct an Arabic questions and answers dataset consists of 300 questions, each accompanied by 5–30 documents, resulting in a total of 6,196 documents.
- Conduct experiments on six LLMs (GPT-4o, GPT-3.5, Claude-4 sonnet, Llama-4, Llama-3, and DeepSeek-V3) to evaluate the ability of LLMs to extract information under different levels of included noisy documents.
- Evaluation of six LLMs on a negative rejection task in which the LLM model is given a large set of documents that lack the correct answer.
- Analyzing the model's errors in both noise robustness and negative rejection tasks and explaining the reasons for those errors.

## 2 Related work

(RAG) (Lewis et al., 2020b) aims to address the problem of LLM out-of-date information and factual hallucinations by retrieving external knowledge documents and incorporating them into the prompt. This enhances LLM effectiveness when responding to complex prompts. It has been widely used in a number of different domains, such as medicine (Chen et al., 2025), law (Li et al., 2025), and education (Zheng et al., 2025). As well as multilingual RAG benchmarks such as MEMERAG (Liu et al., 2025a), which includes five languages (English, German, Spanish, French, and Hindi). In their work, they were motivated by the fact that many other multilingual RAG benchmarks rely on automatic translations, which introduce errors and cultural mismatches into the dataset. A second benchmark, XRAG (Liu et al., 2025b), also includes five languages: English, Arabic, German, Spanish, and Chinese. Their main goal is to assess RAG's ability to retrieve results where the user's language does not match the retrieval result. Another multilingual benchmark, Halluverse25 (Abdaljalil et al., 2025a) provides a framework for measuring hallucinations in multiple languages.

For research that only supports the Arabic Language, Al-Rasheed et al. (2025) evaluates the performance of six semantic embedding models to accurately retrieve text segments that correspond to the input query. Another work by El-Beltagy and Abdallah (2024), did a similar approach in which they present a study on exploring various semantic embedding models in the retrieval stage and several LLMs in the generation stage. In our study, we assessed six LLMs for their performance in answering questions from documents containing noise and irrelevant content. Moreover, we tested LLM's ability to reject questions when the answer was absent in the given documents.

Finding relevant and comprehensive documents that meet the needs of RAG is one of the most challenging tasks. In Trivedi et al. (2023), a solution was proposed where documents are retrieved through multiple reasoning steps. When a user asks a question, such as where the first Camry car was manufactured. Through a series of steps, the RAG system will first retrieve documents related to which manufacturer owns the Camry. Afterwards, they will retrieve documents relevant to the location of this manufacturer. Another solution proposed in Lee et al. (2024), where they eliminate irrelevant sentences from a retrieved document. Then, reconstruct the document to make it more consistent. Another issue is when the answer to the question is not found in the retrieved documents. Peng et al. (2025) argues that many RAG systems do not reject questions if the answer is absent. They conducted a comprehensive study to examine whether LLMs can reject six different types of request that cannot be fulfilled. According to our experiments in this paper, the problem of rejecting questions when no answer is available is also a significant issue in the Arabic language, indicating the need for further research into this problem.

As LLMs evolve, benchmarking has become a reference point where we can compare different models, highlight limitations, and propose solutions to improve the performance of current and future LLMs. Guo et al. (2024) Si et al. (2024) evaluate LLMs in the area of scientific discovery, where they assess their ability to come up with novel research ideas. Almohaimeed et al. (2025a) and Saha and Feizi (2025) Develop benchmarks to test how well LLMs are able to distinguish between articles written by humans and AI-generated articles. In Chen et al. (2024) they have published two benchmarks, one in English and one in Chinese, testing six LLMs in different fundamental abilities that are required by each RAG. A key ability of the LLM is its noise robustness, which makes it capable of answering a question based on a number of documents, even if most of these documents contain irrelevant information. A second ability is negative rejection, where the LLM rejects answering questions if no answer is found in the retrieved documents. Similarly, we were motivated by their research to determine whether the last released LLMs will face a significant challenge for noise robustness and negative rejection. However, we focused our attention on the Arabic language.

## 3 Benchmark

### 3.1 Proposed benchmark

The RAG system mainly relies on the quality of externally retrieved documents and the ability of LLMs to extract information from those documents. In the context of the Arabic language, the Arabic morphology is way more complex than English and it includes hundreds of dialects. Due to this, the challenges that face RAG system on English are more significant when it comes to Arabic language. Among these challenges are the presence of noise, irrelevant information, and misleading statements in documents. Moreover, most RAG systems rely on search engines to extract external documents, and those search engines return results from both reliable and unreliable sources. Consequently, LLMs may struggle to distinguish relevant from irrelevant information, leading to confidently responding with incorrect information. A solution to this kind of hallucination is essential in order to maintain user trust in LLM. Our objective in this paper is to provide a clear understanding of the problem by measuring six LLMs in two essential RAG abilities: noise robustness and negative rejection.

Noise robustness: Measures the ability of the LLM to extract correct information when given a user request and a set of documents (relevant and irrelevant to the request). The assessment will be conducted under five different settings: 0%, 20%, 40%, 60%, and 80%. In all cases, except for 0%, noisy documents will be present, so we analyzed how much the presence of different percentages of noisy documents affects LLM performance.

Negative rejection: Determine whether the LLM has the ability to reject answering questions where the answer does not appear in the given documents. In this case, the assessment setting is 100%, which means that all of the given documents are noise and do not contain the needed information. It is considered correct if the model responds with something like “I am unable to answer the question due to a lack of information.”

### 3.2 Dataset creation

**Figure 2:**
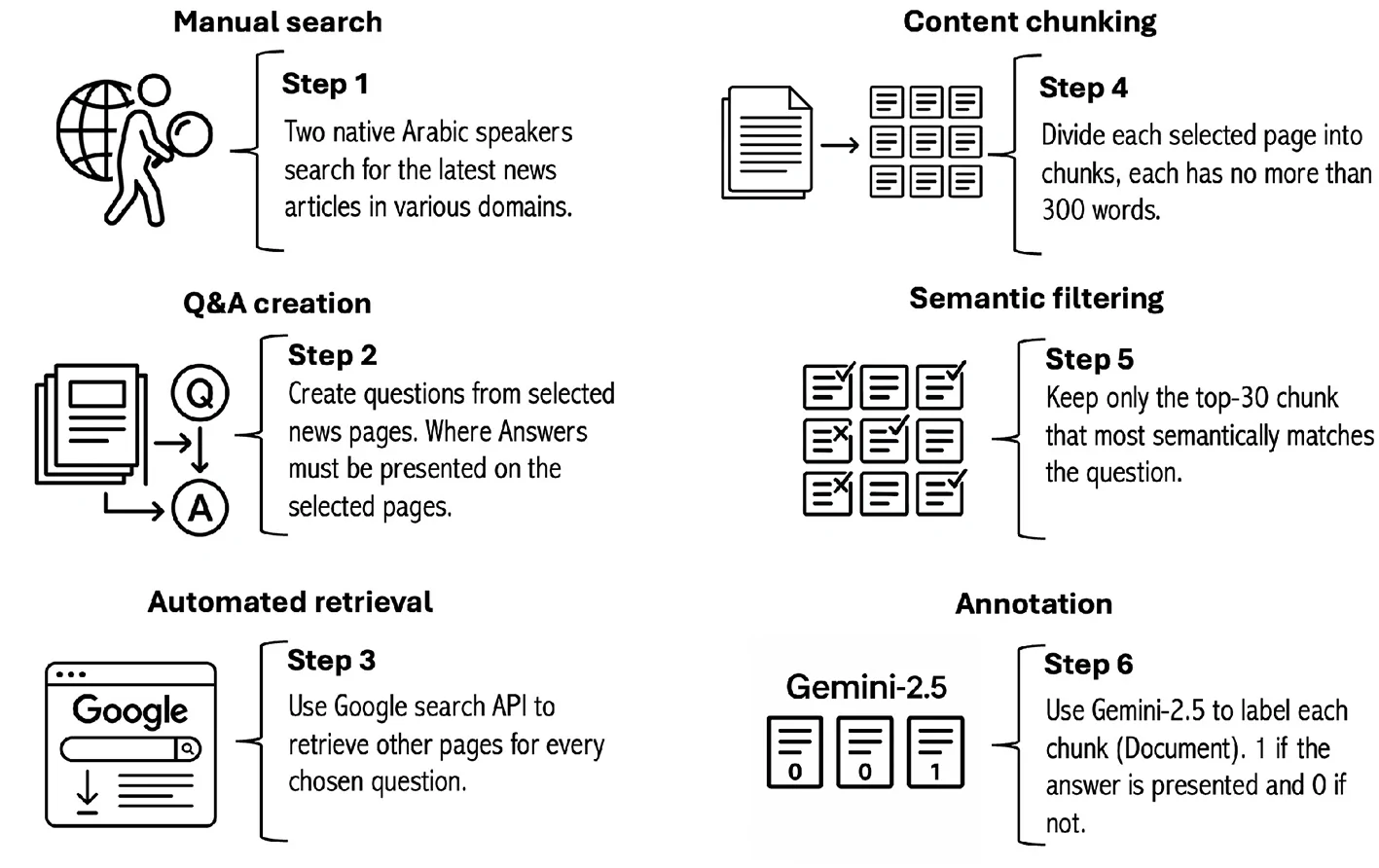
An overview of the data construction and annotation pipeline used to construct the benchmark for evaluating large language models on retrieval-augmented generation (RAG) tasks.

Our approach for generating the data necessary to assess LLMs in RAG tasks is illustrated in Figure 2. First, two native Arabic speakers searched popular Arabic news websites in order to come up with questions related to various domains (politics, sports, business, and others). To ensure that LLMs did not rely on memorized answers from previous exposure to news articles, all questions were based on articles published after the release date of the LLMs used in our experiments.

380 questions were created in steps 1 and 2 from Figure 2, but were later reduced to 300 as many questions were not supported by a sufficient number of online articles. Next, we used Google search API (Google, 2025) to automatically fetch relevant pages for every question. Then each of the relevant pages was divided into chunks of no more than 300 words. Some questions had more than 700 chunks. After that, we use a MiniLM-L12 semantic matching model (Wang et al., 2020) to compare the words in the questions with the words in each segment. The top-30 chunks matching the questions have been selected as external documents. Some questions had a total number of chunks of less than five; these questions were eliminated.

Regarding the annotation process, documents that contain the answer will be labeled as 1, while documents not containing the answer will be labeled as 0. To do this task, Gemini-2.5 (Google DeepMind, 2025) was given the question, the answer to the question, and a document of approximately 300 words. Then, ask it to determine whether the given answer is presented in the document either directly or by using multiple reasoning steps. If yes, respond with 1 and if not, respond with 0. The number of documents that have been automatically annotated is 6,196. To ensure that Gemini-2.5 (Google DeepMind, 2025) work is reliable, we randomly selected 500 documents out of 6,196 and checked the quality of the annotations provided by Gemini-2.5. There were no errors found. This does not imply that all 6,196 are correct, but it indicates that they are suitable for assessing LLMs with respect to noise robustness and negative rejection. We ended up with 300 questions, each with external documents labeled as relevant, where the answer is presented, and noisy if the answer is not presented.

Data were collected using the same strategy used in most RAG benchmarks, which is based on search engines to obtain relevant documents. However, in order to ensure diversity, the questions were derived from a variety of domains and no more than three questions were based on a single news article. Moreover, for more natural-sounding questions, all questions have been written by two native Arabic speakers, and not all documents have been included, as we have just selected the top 30 documents that semantically match the question.

## 4 Experiments

### 4.1 Evaluation metrics

In order to evaluate the Arabic RAG benchmark, we used two metrics. The first is the accuracy for evaluating LLM performance in the noise robustness task. The objective of the task is to determine the number of correct answers obtained by the LLM for all the questions. We use accuracy because each question has a single ground-truth answer and is evaluated as correct or incorrect, Therefore, accuracy is sufficient to assess the LLM ability to extract the correct information from noisy documents.

There are two steps in this evaluation process. The first step was to use GPT-4o (Hurst et al., 2024) as a verifier, in which it compares the answer given by other LLM with the ground truth answer. It will respond with 1 if the answer is correct and 0 otherwise. The second step involves a human verifier who will review the work done by GPT-4o (Hurst et al., 2024) and correct any mistakes. The GPT-4o results were accurate in most cases; however, in some cases, it failed, and that is why we rely on the assistance of human verifiers to ensure that the evaluation scores are accurate. To do that, two professors of Computer Science independently reviewed the evaluation results. Evaluation involved comparing each model-generated answer with the corresponding ground truth answer. An answer was considered correct if it conveyed the same meaning as the ground truth, regardless of minor differences in wording or sentence structure. Since the evaluation criteria focused on semantic equivalence rather than exact textual matching, the reviewers were concerned about whether the generated response maintained the intended meaning. Each reviewer evaluated approximately half of the evaluation samples.

As an important note, in this setup, GPT-4o serves as one of the models evaluated in the noise robustness task. Then, as a verifier who checks that the model-generated answer matches the ground truth answer. The use of GPT-4o in two roles does not pose a concern due to the fundamental difference between both roles. For the noise robustness task, GPT-4o is given the question and the retrieved documents as input, then asked to generate an answer. However, when GPT-4o is used as a verifier, it is provided with only two texts, the ground-truth answer and the model-generated answer, without access to the question, the retrieved documents, or the name of the evaluated model. Then, it is asked whether the model-generated answer contains the correct answer or gives an equivalent meaning, producing a binary score of 1 for correct answers and 0 for incorrect answers. Due to this, the inputs, outputs, goals, and prompt instruction differ substantially between these two roles, effectively minimizing the possibility of evaluation bias.

The second evaluation metric is the rejection rate, which is related to the negative rejection task, in which we evaluate how well the LLM rejects answering questions when the answer does not appear in the given documents. An evaluation is performed manually by a member of our team, who checks all the answers provided by the LLMs. If the model responds with “I cannot answer the question due to insufficient information” or a similar response, it is considered to be correct; otherwise, it is considered to be incorrect. Our goal in using rejection rate is not to measure answer quality, but instead to determine whether the model is able of correctly identifying the absence of supporting information in the provided documents and refusing to answer. Each instance has only two possible outcomes, successful rejection or unsuccessful rejection. Therefore, the rejection rate is a direct and interpretable measure for this task.

### 4.2 Settings

Our experiments evaluate six large language models (LLMs) including GPT-4o (Hurst et al., 2024), GPT-3.5 (OpenAI, 2024), Llama-4 (Meta AI, 2025), Llama-3 (AI, 2024), Claude-4 sonnet (Anthropic, 2025b), and DeepSeek-V3 (DeepSeek-AI et al., 2024). Before any experiments, we applied some settings to ensure a fair comparison between those LLMs. First, for every question, it was accompanied by 5 documents. The number of relevant and noisy documents included is dependent on the percentage level set beforehand. While the benchmark contains 6,196 annotated documents, the number of documents associated with each question ranges from 5 to 30. Hence, the benchmark is more of a document pool than a fixed evaluation context. When the noise percentage setting is set to 0%, all five documents have the correct answer. Under the 20%, 40%, 60%, and 80% noise settings, the document set consisted of 4 relevant and 1 noisy documents, 3 relevant and 2 noisy documents, 2 relevant and 3 noisy documents, and 1 relevant and 4 noisy documents, respectively. Having a larger document pool than required for evaluation ensured that documents were selected randomly and minimized the risk of selection bias. In order to ensure reproducibility, fixed random seeds were used during document sampling, with the same document sets being presented to all LLMs under each noise level. This design ensures a fair comparison among models and eliminates any bias resulting from different selections of documents.

To support reproducibility, the exact Application Programming Interface (API) model identifiers, inference platforms, and experiment run dates have been documented. GPT-4o and GPT-3.5 were accessed via the OpenAI (OpenAI, 2025) platform, while Claude-4 sonnet, Llama-4, Llama-3 and DeepSeek-V3 were accessed through the DeepInfra platform (DeepInfra, 2025) using their corresponding model identifiers. To ensure that identical document sets were used across all noise levels and for all LLMs evaluated, we fixed the random seed to 42. For each model, the temperature was set to 0 to minimize generation randomness and ensure repeatable results. Context windows for each model were configured to accommodate the complete prompt and retrieved documents without truncation. Decoding parameters were left at their default settings with no additional sampling controls applied. See our Github page^1^ for more details.

**Figure 3:**
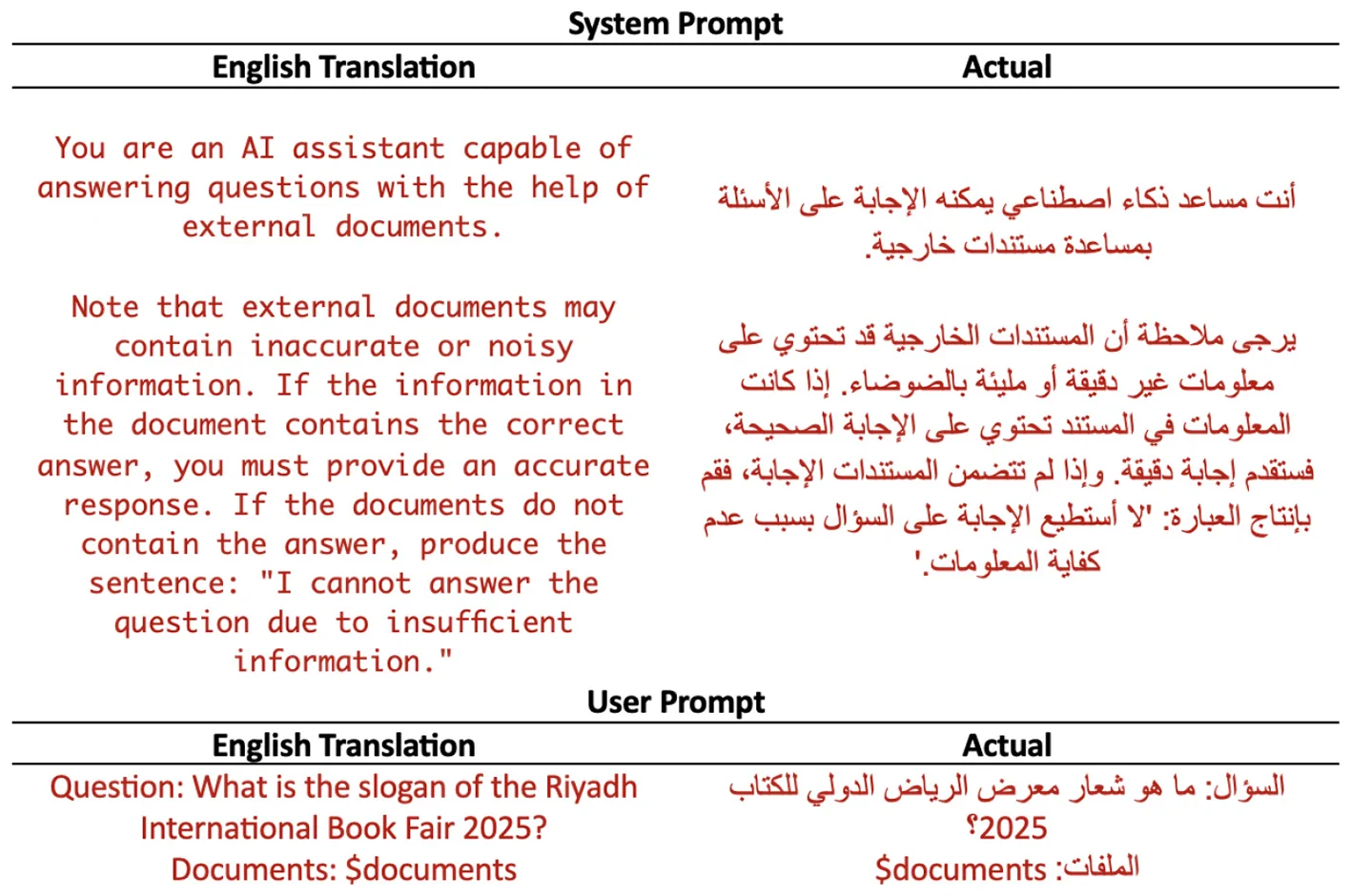
An example of the system and user prompt used to prompt all six large language models. On the right side of the system prompt are Arabic sentences, and on the left side is the English translation. System prompts will limit the LLM to acting in accordance with its instructions. Following that is the user prompt, which contains the user question and external documents.

Additionally, the same prompt template was used across all models, including: system prompt and user prompt. As seen in Figure 3, the system prompt will give the LLM a task to act on. Moreover, it will restrict the LLM to answer the question using only three choices, the correct answer, documents have insufficient information, or there is some contradiction in the output. On the other hand, for the user prompt, the LLM will get only the question and the external documents. All the six models answers strictly follow the instruction, and none of them has generated content out of the aforementioned three options. To avoid bias, no restriction has been applied to any model alone. Moreover, we make sure to choose the external documents randomly to remove any bias from our side. Additionally, at a certain noise level, such as 60%, the questions and documents presented to a model are identical to those presented to all other models.

### 4.3 Results

#### 4.3.1 Noise robustness

From Table 1, we can see the evaluation of six LLMs with different percentages of noise documents. For 0% settings, when there are no noise documents included, the best model is Claude-4 sonnet (Anthropic, 2025b). It has achieved 91.67%, showing remarkable results in Arabic. Claude-4 sonnet is well known for its success in Arabic tasks, as outlined in Almohaimeed et al. (2025a) and Attia et al. (2025). Anthropic (2025a) reveals that Claude-4 sonnet achieves 96% on several Arabic benchmarks using zero-shot and chain-of-thought prompting. The given percentage is relative to the performance of Claude-4 sonnet in English benchmarks, which means that its performance in English decreases by only 4% when handling Arabic tasks, highlighting its capability and strength in the Arabic language. Despite the fact that the reported scores show robust Arabic capabilities, they do not represent a universal 4% performance loss across all Arabic tasks.

**Table 1:** Performance of LLMs under different noise document percentages.

Model	Noise document percentage				
	0%	20%	40%	60%	80%
GPT-4o	74.33	74.00	72.67	72.67	61.00
GPT-3.5	80.00	77.33	77.00	73.67	58.67
Claude-4 Sonnet	**91.67**	**91.67**	**91.33**	**92.33**	**87.00**
DeepSeek-V3	86.00	85.33	83.67	81.67	68.00
Llama-3	80.33	80.67	79.67	77.33	69.33
Llama-4	73.33	72.00	72.67	72.33	65.00

Bold values indicate the best performance model.

In second and third place, DeepSeek-V3 (DeepSeek-AI et al., 2024) achieved 86% and Llama-3 (AI, 2024), achieved 80.33%. Again, Claude-4 sonnet strength is evident as the differences between the first and third LLM are approximately 11%. Another note, the performance of the GPT-3.5 (OpenAI, 2024) is still acceptable, as it obtained an average score of 80%. However, in many other investigations, such as (Almohaimeed et al., 2025b) and Almohaimeed et al. (2024b), GPT-3.5 has always been unable to master the Arabic language compared to other LLMs. Unexpectedly, GPT-4o and Llama-4 achieved the lowest performance in noisy documents 0%, 74.33%, and 73.33%, respectively. The reason for this is that most of the questions that have not been answered correctly are answered with “I am not able to answer the question due to insufficient information.” This does not mean that the LLM cannot answer the question. However, it implies that if there are any ambiguities in the given documents, GPT-4o (Hurst et al., 2024) and Llama-4 (Meta AI, 2025) will act more carefully, which will make them respond with greater caution.

Also, in Table 1, we examine the performance of the six LLMs under different levels of noise, 20%, 40%, 60%, and 80%. It can be seen that the performance of Claude-4 sonnet remains the best, as it has not been adversely affected by documents with noise levels of 20%, 40%, and 60%. However, it decreased by 4.67 percentage points when the percentage of noisy documents reached 80%. Even though it performed very well, the results illustrate that there is still some room for improvement in terms of the performance of Claude-4 sonnet. Similar results have been observed in GPT-4o, as it has not had a significant impact on noisy documents in the levels of 20%, 40%, and 60%. However, when 80% is chosen, performance decreases by approximately 13%, resulting in an accuracy of 61%.

On the other hand, when considering all other models, Llama-4, Llama-3, DeepSeek-V3, and GPT-3.5, we can observe a gradual decline in performance as the noise document level increases. As an example, DeepSeek-V3 achieved 86% under 0%, decreased by 0.67% at 20%, 2.33% at 40%, 4.33% at 60%, and 18% at 80%. In addition, it should be noted that under 80% the performance of all six LLMs decreases significantly. Most likely because only one of the five documents provides the correct answer, whereas the remaining four documents are noisy, thus hindering the LLM from identifying and relying on relevant information.

#### 4.3.2 Negative rejection

For the second task, negative rejection, Table 2 shows that GPT-4o (Hurst et al., 2024) and Llama-4 (Meta AI, 2025) achieve very good performance compared to DeepSeek-V3 (DeepSeek-AI et al., 2024), Llama-3 (AI, 2024), and GPT-3.5 (OpenAI, 2024). It supports our argument that GPT-4o and Llama-4 perform better than DeepSeek-V3, Llama-3, and GPT-3.5, but respond in a safe manner rather than answering the question with less confidence. GPT-4o and Llama-4 scores, respectively, are 80.67% and 85.67%. They both perform well when it comes to rejecting answers to questions when the answer is not included in the external documents. On the other hand, Claude-4 sonnet (Anthropic, 2025b) achieved the best results with 90.67%, a significant lead over Llama-4. DeepSeek-V3 score is 75.67%, while the Llama-3 score is 78.67%. Both show acceptable results. However, there is still a lot to be improved compared to more advanced and larger LLM models. In terms of GPT-3.5, it performed very poorly, achieving only 69.33%. Despite the fact that the answer was not presented in the external documents, GPT-3.5 answered the question with high confidence. This is a high rate of hallucination and will affect the LLM industry negatively, as some users will lose trust in LLM and others will base their actions on incorrect information.

**Table 2:** Model performance under 100% noisy documents.

Model	100% Noisy documents
GPT-4o	80.67
GPT-3.5	69.33
Claude-4 sonnet	**90.67**
DeepSeek-V3	75.67
Llama-3	78.67
Llama-4	85.67

Bold values indicate the best performance model.

#### 4.3.3 Confidence interval analysis

For each reported result, we compute non-parametric 95% bootstrap confidence intervals. A bootstrap resampling was conducted using 10,000 resamples over 300 questions. Confidence intervals relate to the 2.5th and 97.5th percentiles of the bootstrap distribution.

Table 3 illustrates that Claude-4 sonnet achieves the highest accuracy at all noise levels. Additionally, bootstrap confidence intervals for Claude-4 sonnet consistently show margins of around 3%. In contrast to most other models, which have margins of about 5%. This indicates lower sampling variability and more precise predictions. It is also noteworthy that for each model, the interval widths change only slightly as the percentage of noisy documents increases. As a result, it appears that increasing document noise percentage has a greater influence on LLM performance than the degree of uncertainty associated with LLM scores. Furthermore, the confidence interval margins of all LLM are consistent with an expected level of uncertainty for a benchmark comprising 300 evaluation questions. Thus, providing additional confidence in the reliability of the reported results.

**Table 3:** Estimated accuracy and rejection rate with 95% bootstrap confidence intervals.

Model	0%	20%	40%	60%	80%	100%
GPT-4o	74.33 (+5.00/–5.00)	74.00 (+5.00/–5.00)	72.67 (+5.00/–5.00)	72.67 (+5.00/–5.00)	61.00 (+5.67/–5.67)	80.67 (+4.33/–4.34)
GPT-3.5	80.00 (+4.67/–4.67)	77.33 (+4.67/–4.67)	77.00 (+4.67/–4.67)	73.67 (+5.00/–5.00)	58.67 (+5.67/–5.67)	69.33 (+4.00/–4.00)
Claude-4 Sonnet	91.33 (+3.00/–3.33)	91.33 (+3.00/–3.33)	91.67 (+3.00/–3.00)	92.00 (+3.00/–3.33)	86.67 (+3.67/–4.00)	90.67 (+3.10/–3.20)
DeepSeek-V3	86.00 (+3.67/–4.00)	85.33 (+4.00/–4.00)	83.67 (+4.00/–4.33)	81.67 (+4.33/–4.33)	68.00 (+5.33/–5.33)	75.67 (+4.66/–5.00)
Llama-3	80.33 (+4.33/–4.67)	80.67 (+4.33/–4.67)	79.67 (+4.67/–4.67)	77.33 (+4.67/–5.00)	69.33 (+5.00/–5.33)	78.67 (+4.66/–4.67)
Llama-4	73.33 (+5.00/–5.00)	72.00 (+5.00/–5.00)	72.67 (+4.67/–5.00)	72.33 (+5.00/–5.00)	65.00 (+5.33/–5.33)	85.67 (+4.66/–5.00)

The values are reported as an estimate (+upper margin/–lower margin). The first five columns correspond to the noise robustness task and the 100% column to the negative rejection task.

#### 4.3.4 McNemar test analysis

In order to determine whether the observed performance differences were statistically significant, a pairwise McNemar test with Holm correction was conducted across all six models. The results consistently demonstrated the superiority of Claude-4 Sonnet over the lower-performing models. At 0% noise level, Claude-4 sonnet demonstrated significant improvements over Llama-4, GPT-4o, GPT-3.5, and Llama-3, while its comparison with DeepSeek-V3 was non-significant. In similar situations, Claude-4 Sonnet maintained noticeably better performance than the four competing models at a 20% noise level, while DeepSeek-V3 performed significantly better than GPT-4o and Llama-4. By increasing the noise level to 40% and 60%, Claude-4 Sonnet demonstrated significant performance advantages over all five models, while DeepSeek-V3 also demonstrated significant performance advantages over GPT-4 and Llama-4. At the most challenging 80% noise level, the largest performance differences were observed, with Claude-4 Sonnet significantly outperforming every other model by 17.3–28.0 percentage points. Additionally, both DeepSeek-V3 and Llama-3 significantly outperformed GPT-4o and GPT-3.5, which suggests that they are more robust when documents are extremely noisy. Overall, the McNemar analysis confirms that the ranking observed from accuracy scores is statistically reliable and that performance differences become more noticeable as the proportion of noisy documents increases.

## 5 Discussion

The performance of LLMs was generally good, with the exception of a few errors when there were no noisy documents included. In contrast, if we include noisy documents, we can observe a drop in performance on all of them, especially when the noisy documents represent 80% of all external documents. It is necessary to examine the incorrect answer manually and look for reasons why the performance has dropped. Having done that, we have discovered that most of the errors are caused by one of the following three points.

### 5.1 Over-caution to avoid hallucinations

The percentage of hallucinations was very high when LLMs were introduced, but with the evolution of LLMs, some efforts have been made to prevent LLM hallucination. Unfortunately, this has some consequences, as shown in Table 4. In the first and second examples, the answer was clearly visible to Llama-4 (Meta AI, 2025), but it responded “I am unable to provide an answer because I do not possess sufficient information.” Meanwhile, GPT-4o (Hurst et al., 2024) responded correctly to the same questions and documents. The consequences of such over-cautiousness include preventing the full power of the LLM reasoning ability from being employed. Over-cautious situations occur when the LLM is not 100% certain that the answer they have is correct, especially in multilingual or non-English situations. Consequently, when the model detects any ambiguity, it may choose the safe response and respond with “I do not have enough information to provide an answer.”

**Table 4:** Examples of RAG errors across LLMs.

Question	Answer	Supporting document excerpt	Model	Error type
In which province did the innovation capable of treating fractures and fixing bone fragments in a very short period of time, developed in late 2025, emerge?	Zhejiang	A research team in Zhejiang Province in eastern China announced the development of a new “bone glue” capable of treating fractures and fixing bone fragments in just 3 min, described as a scientific breakthrough in orthopedic surgery. According to NDTV, the innovation is called Bone-02.	Llama-4	Over-cautious
Who is the French goalkeeper who delivered a legendary performance in their match at Roma's stadium in the Europa League on October 2, 2025?	Barky Ozair	Roma suffered a major shock in the Europa League after losing 1–0 to Lille, in a match that witnessed a legendary performance by Barky Ozair, Lille's goalkeeper.	Llama-4	Over-cautious
In which country was it announced that the new government would include an AI-powered minister in 2025?	Dila	Albanian Prime Minister Edi Rama announced that the new government will include an “AI-created minister” responsible for managing public funding projects and combating corruption. He added that “Dila” (meaning “sun” in Albanian) will be a cabinet member without a physical presence, created virtually using artificial intelligence.	GPT-3.5 & Llama-3	Noise dominance
Which Italian cities will host the 2026 Winter Olympics?	Milan and Cortina	Sofia Goggia and Arianna Fontana expressed excitement about the Milan–Cortina 2026 Winter Olympics, highlighting that competing at home is a rare opportunity and discussing what makes the Games special.	GPT-3.5 & Llama-3	Noise dominance
What percentage did a World Bank executive say in September 2025 would be allocated to Africa?	70%	Business Insider Africa quoted Axel van Trotsenburg stating that around 45% of the World Bank's resources are directed to Africa, while 70% of concessional loans are allocated to Africa alone.	Llama-3	Long-distance information
What is the start date of the ADIPEC 2025 exhibition and conference?	November 3	ADIPEC 2025 will bring together global energy leaders, including more than 45 ministers and 250 CEOs. Hosted by ADNOC, it will take place from November 3 to 6.	GPT-3.5	Long-distance information

The Question is a translation of an Arabic question from our dataset. The Answer is the ground-truth answer. The Supporting Document provides an excerpt from an external document containing the answer. The Model is the LLM that answered incorrectly. The Error Type refers to one of the three error categories discussed in Section 5.

### 5.2 Noise dominance from irrelevant documents

Another challenge that RAG systems face is that in addition to documents that contain the correct answers, many noisy and irrelevant documents are presented as well. Even though the answer may sometimes be explicitly stated in one document, the model may ignore it because some of the noisy documents have a higher semantic similarity to the question than the relevant document. Additionally, in some circumstances, when there are a large number of noisy documents, LLM may choose to use an answer from one of them as a basis for its response. The risk of this kind of situation is usually greater for smaller LLMs. In Table 4, the fourth example asks: “Which Italian cities will host the 2026 Winter Olympics?” Both GPT 3.5 (OpenAI, 2024) and Llama-3 (AI, 2024) add the city of Turin. The reason for this mistake is that both models confuse who bid to host the Olympics with who actually hosted them. Another example, the third question in Table 4, which is “In which country was it announced that the new government would include an AI-powered minister in 2025?”, GPT 3.5 and Llama-3 was unable to answer the question, even though it appeared multiple times, as other noisy documents distracted GPT 3.5 from the specific case of Albania. GPT 3.5 and Llama-3 answer both the third and fourth questions in Table 4 correctly, when less noisy documents are included. However, when the level of noise increased, they became diverted from the correct answers, which led to wrong answers.

### 5.3 Long distance information

LLMs sometimes fail to correctly answer questions when the answer in the given documents is far from the question-related words. The problem is not unique to Arabic as it has been discussed in other RAG papers as Chen et al. (2024). For these types of questions, the question-related semantic words are usually presented in a specific location within the document, while the answer is located at a far distance. The fifth and sixth examples in Table 4 illustrate this. In both cases, the words related to the question are presented at the beginning of the external document, while the answers are presented at the end. Thus, this document may be ignored by the LLM, and other irrelevant documents may be given a higher priority.

## 6 Limitation

### 6.1 Categorization of noisy documents

In our work, we define noisy documents as those which do not contain the correct answer to a given question. These noisy documents influence LLM responses in a number of ways. Based on our manual error analysis, some errors are caused by noisy documents that contain words or phrases that have a high degree of lexical or semantic similarity to the question, thus making them appear relevant despite the absence of a correct answer. Other errors are caused by the large amount of irrelevant context introduced by noisy documents. Additionally, some errors are a result of long-distance information, where the answer lies far from the content related to the question, making it more difficult to locate the correct answer.

Consequently, the behavior of a model may be affected differently by different types of noise. Although our qualitative error analysis illustrates the importance of these distinctions, a more detailed categorization of noise documents was beyond the scope of this study. An analysis of this type would require a considerable amount of manual annotation, as each document would need to be carefully reviewed and classified in accordance with one or more noise categories. In addition, noisy documents do not always fall into a single category; they may exhibit lexical overlaps, semantic similarities, and long-distance information simultaneously, making the annotation process more challenging. Our main objective in this work was to establish a benchmark and provide an initial assessment of current LLM capabilities in the Arabic language, specifically noise robustness in RAG. Developing a more detailed analysis of noise types and their impact on different LLMs lies beyond this work and represents an important research area in the future.

### 6.2 Retrieval stage

The focus of this study is on the generation component of the RAG pipeline, rather than evaluating the entire pipeline. While retrieval techniques such as retrieving documents using a search engine and calculating semantic matching scores using MiniLM-L12 model (Wang et al., 2020) were used to collect candidate documents during benchmark construction, standard retrieval metrics were not used to evaluate the retrieval component. Instead, relevant and noisy documents were manually controlled in the experiment in order to achieve predefined noise percentages. The choice of this approach was intentional, as the primary objective of this study was to assess the noise robustness and negative rejection capabilities of the generator component in Arabic RAG systems. Accordingly, the reported results should be interpreted as an assessment of generator stage robustness rather than the performance of the entire RAG system. In the future, this benchmark may need to be extended to include retrieval evaluation and reporting standard retrieval metrics such as Precision@k, and Recall@k. Furthermore, it is necessary to investigate Arabic-specific embedding models and their impact on document selection, as a number of studies (El-Beltagy and Abdallah, 2024; Almohaimeed et al., 2024a) have demonstrated that choosing an embedding model influences semantic matching and retrieval performance in Arabic NLP tasks significantly.

## 7 Conclusion and future works

Retrieval-Augmented Generation plays a vital role in preventing hallucinations associated with LLMs. RAG incorporates internal knowledge from the LLM with external, up-to-date information when answering a user question. In this paper, we evaluate the performance of six state-of-the-art LLMs that support Arabic, based on two fundamental RAG abilities, Noise robustness and negative rejection. For the purpose of this evaluation, an Arabic benchmark of 300 questions has been constructed. Results indicate that each LLM has its own issues as GPT-4o, and Llama-4 need to reduce their over-cautionary behavior, which prevents them from answering questions even if the answer is obvious. Smaller models, such as GPT-3.5 and Llama-3, perform worse on both tasks. DeepSeek-V3 scores relatively well, and Claude-4 sonnet achieve the highest performance, but there are still significant gaps that need to be addressed by future research.

### 7.1 Future works

To the best of our knowledge, this paper is the first Arabic paper that discusses the impact of noisy documents on RAG systems in Arabic. Many Arabic future research directions can be drawn from this paper. A possible direction is to evaluate the RAG systems on tasks where factual errors are presented within external documents. In our study, all of the documents either contain the answer or do not, but they do not contain any actual errors. The evaluation of the LLM ability to reason over multiple documents in which the correct answer appears multiple times, but some documents contain misleading information is crucial. Having this capability is important since many online sources used by LLMs contain incorrect information.

Another direction may be to test the RAG systems on multi-hop reasoning, where the answer is not clearly presented in the documents and requires reasoning steps to reach it. For example, when a question asks about a person's role at the time of receiving an award, one paragraph may mention the name of the individual who won the award, another paragraph may describe that individual's career path, and a separate paragraph may describe the role that individual was holding during that period. This kind of question requires a connection between different facts in the given document to identify the correct answer. It is an important ability, as most of the questions in our benchmark do not require much reasoning, and the answers are mostly explicit.

Additionally, there is also another direction related to a task named information integration, which requires answers to be derived from multiple documents rather than just one. As an example, answering a question regarding a company's total revenue in a given year may require the summation of quarterly revenues reported across multiple documents, each covering a different quarter of the year. The benefit of this ability is that it allows the LLM to have more information than the actual news articles, and it allows the LLM to extract new data that has not been reported previously.

## Data Availability

The raw data supporting the conclusions of this article will be made available by the authors, without undue reservation.

## Footnotes

1
Github.com/Saleh-Almohaimeed/Arabic-RAG-Benchmark
